# Dietary pattern scores in relation to pre-diabetes regression to normal glycemia or progression to type 2 diabetes: a 9-year follow-up

**DOI:** 10.1186/s12902-023-01275-9

**Published:** 2023-01-20

**Authors:** Parvin Mirmiran, Shabnam Hosseini, Zahra Bahadoran, Fereidoun Azizi

**Affiliations:** 1 Nutrition and Endocrine Research Center, Research Institute for Endocrine Sciences, Shahid Beheshti University of Medical Sciences, No 24, A’rabi St, Yeman Av, P.O. Box, Velenjak, Tehran, 19395-4763 Iran; 2 Department of Clinical Nutrition and Dietetics, Faculty of Nutrition Sciences and Food Technology, National Nutrition and Food Technology Research Institute, Shahid Beheshti University of Medical Sciences, Tehran, Iran; 3 School of Human Nutrition, Faculty of Agricultural and Environmental Sciences, McGill University, Montreal, QC Canada; 4 Endocrine Research Center, Research Institute for Endocrine Sciences, Shahid Beheshti University of Medical Sciences, Tehran, Iran

**Keywords:** Western diet, Mediterranean diet, Pre-diabetes, Dietary pattern, Progression, Regression

## Abstract

**Background:**

We aimed to assess potential associations of habitual dietary pattern scores in relation to the risk of pre-diabetes (Pre-DM) progression to type 2 diabetes mellitus (T2DM) or the chance of returning to normal glycemia.

**Methods:**

This cohort study included 334 Pre-DM individuals (mean age of 49.4 years, and 51.5% men) who participated in the third phase of the Tehran Lipid and Glucose Study (2006–2008) and followed up for a median of 9 years. A validated food frequency questionnaire at baseline assessed usual intakes of the participants. Major dietary patterns were identified using principal component analysis. The DASH score and Mediterranean diet score (MDS) were also calculated. Multinomial logistic regression analysis was used to estimate the odds ratios (95% confidence intervals (CIs)) of developing T2DM and returning to normal glycemia in relation to dietary pattern scores.

**Results:**

During the study follow-up, 39.8% progressed to T2DM, and 39.8% returned to normal glycemia. Three following major dietary patterns, including Western-style (with a higher load of red meats, hydrogenated fats, sodium, and total fat intakes), healthy pattern (with a higher load of whole grains, vegetables, and dairy products), and processed-foods pattern (with a higher load of processed-meats, fast-foods, salty snakes, and sweets and candies) were identified. The Western-style dietary pattern increased the risk of progressing to T2DM by 38% (OR = 1.38; 95% CI = 1.00 to 1.89, *P* = 0.050). Other dietary pattern scores were not related to regression or progression from Pre-DM.

**Conclusion:**

The Western-style dietary pattern (characterized by higher load of red meats, hydrogenated fats, sodium intake, and high-GI foods) may accelerate the progression of Pre-DM to T2DM.

**Supplementary Information:**

The online version contains supplementary material available at 10.1186/s12902-023-01275-9.

## Background

Prediabetes (Pre-DM) is a transitional glycemic state (between normal glucose regulation and type 2 diabetes mellitus (T2DM)) which may progress to T2DM or regress to normal glycemia [1]. The Pre-DM state is associated with a significantly higher risk of developing T2DM [1, 2]. The global prevalence of Pre-DM is increasing quickly [3, 4], and it is estimated that 587 million individuals will live with Pre-DM by 2045 [5], with an annual progression rate of 5–10% to T2DM [6]. Previous studies show that lifestyle modifications (i.e., diet and physical activity) can prevent or even revert the Pre-DM progression [6, 7].

In addition to medical treatments [8] and intensive training programs [9, 10], dietary modifications have been effective to attenuate the risk of Pre-DM progression to T2DM [11–13]. Dietary patterns like Mediterranean Diet (Med Diet) [14] affect the risk of Pre-DM [15, 16]. However, the effect of dietary intervention per se on the progression towards T2DM is still controversial [9]; a combined-approach (dietary intervention + lifestyle modifications) was reported to be successful to preventing or delaying the progression from Pre-DM to T2DM [17–20]. Observational studies also reported adherence to Med Diet [21, 22], higher intake of low-fat dairy [23], and a reduced dietary glycemic load (GL) [24] may reduce risk of progressing to T2DM among subjects with Pre-DM. The possible association of habitual dietary patterns with Pre-DM regression to normal glycemic regulation (NGR) is less investigated; some evidence indicate that lifestyle interventions might have the power to convert back the Pre-DM state to NGR [25, 26], especially in women [7].

Here, we aimed to examine the association of dietary patterns scores with Pre-DM progression and regression in an Iranian population. Using dietary patterns to assess the diet-disease relationship is a more comprehensive and holistic approach which gives a better view of interactive effects of dietary components [27].

## Methods

### Study population

This longitudinal study was conducted in the framework of an ongoing community-based prospective study (the Tehran Lipid and Glucose Study, TLGS), which started in 1999 with 15,005 individuals, aged ≥ 3 years, to investigate and prevent non-communicable diseases [28]. For the current study, we recruited adult (age ≥ 21 y) Pre-DM men and women (*n* = 334) with completed data (usual diet, demographics, anthropometrics, and biochemical measurements) participated in the third phase of the TLGS (2006–2008) and followed up to the sixth phase of the TLGS (2015–2017). The study protocol was carried out according to the relevant guidelines expressed in the Declaration of Helsinki.

Written informed consent was obtained from all participants. The ethics research council of the Research Institute for Endocrine Sciences, Shahid Beheshti University of Medical Sciences, Tehran, Iran, approved the study protocol (Ethics code: IR.SBMU.ENDOCRINE.REC.1401.020).

### Demographic, anthropometric and biochemical measurements

Details of data collection and measurements of the variables in the TLGS have been reported elsewhere [28]. In brief, anthropometric data, including body weight, height, and waist circumference (WC) were collected using standard methods. Body mass index (BMI) was calculated as weight (kg) divided by square of height in meters (m^2^). Systolic (SBP) and diastolic (DBP) blood pressures were measured using a standard mercury sphygmomanometer calibrated by the Institute of Standards and Industrial Research of Iran [29]. Blood pressure was measured twice on participants' right arm, after a 15-min rest in a sitting position, with at least a 30-s interval between two measurements. The mean of the two measurements was considered as the participant’s blood pressure.

Details of biochemical measurements in the TLGS samples have been described elsewhere [30]. In brief, measurements of fasting serum glucose (FSG), triglyceride (TG), and high-density lipoprotein cholesterol (HDL-C) levels were all done after a 12- to 14-h overnight fasting. The standard oral glucose tolerance test (OGTT) was performed for all adults (age ≥ 21 y) who were not on glucose-lowering medications.

The physical activity was assessed using the Modifiable Activity Questionnaire (MAQ); the frequency and time spent on light, moderate, hard, and very hard intensity activities according to the list of everyday activities of daily life over the past year were documented and physical activity levels expressed as metabolic equivalent hours per week (MET-hour/week) [31]. Reliability and validity of the Persian version of the MAQ have previously been investigated [32].

### Dietary assessment and dietary pattern scores calculation

The usual dietary intakes of the participants over the previous year were assessed using a validated semi-quantitative 168-item Food Frequency Questionnaire (FFQ) (supplementary file 1). Details of dietary assessment in the TLGS were described elsewhere [33]. In brief, the frequency of food items consumed during the past year was asked daily, weekly, or monthly. Portion sizes of consumed foods reported in household measures were converted to the gram. Since the Iranian Food Composition Table is incomplete and has limited data on raw foods and beverages' nutrient content, the US Department of Agriculture Food Composition Table was used [34]. To remove confounding effect of under- or over-report of energy intakes on estimated-intakes of food groups and nutrients, a residual adjustment was performed using a regression model (with total caloric intake as the independent variable and food/nutrients intake as the dependent variable) [35].

The Dietary Approaches to Stop Hypertension (DASH) diet score was calculated based on the index developed by Fung et al. [36], comprised of 8 components (7 food groups, i.e., fruit + fruit juice, non-starchy vegetables, low-fat dairy products, whole grains, and nuts + seeds + legumes, and one nutrient, i.e., sodium). The DASH score was calculated for each participant; component score for fruit, vegetables, nuts and legumes, low-fat dairy products, and whole grains was the intakes’ quintile ranking (e.g., quintile 1 is assigned 1 point and quintile 5.5 points). For sodium, red and processed meats, and sweetened beverages, where their lower intakes was desired, the lowest quintile was given a score of 5 points and the highest quintile, 1 point. Finally, the component scores were summed up to obtain an overall DASH score ranging from 8 to 40.

Adherence to Med Diet was assessed using a modified version of Trichopoulou’s Mediterranean diet score (MDS) [37, 38], calculated based on nine items, i.e., dietary monounsaturated-to-saturated fats ratio (MUFAs to SFAs ratio), and intakes of whole grains, fruits, legumes, nuts, fish, processed and red meats, and vegetables; for religious reasons, alcohol was excluded from the final score in our study. For each of the components which were hypothesized to be beneficial to health, 1 point was given to a participant if intake was above the median, 0 otherwise. Scores for all eight items were hence summed up to calculate MDS (ranged from 0 to 8).

To obtain major dietary patterns among our population and estimate dietary pattern scores for study participants, principal component analysis (PCA, i.e., a commonly used method for deriving major dietary patterns in population based studies) was used with Varimax rotation based on 15 food items (i.e., whole grains, refined grains, legumes, red meats, fruits and dried fruits, vegetables, total dairy products, fish and poultry, salty snacks, sweets and candies, hydrogenated fats, tea and coffee, total fat and sodium intakes). Three factors were created regarding the scree plot and eigenvalues more than 1. To include in the dietary patterns, we selected the food items with factor loading ≥ 0.30. In our models, all food items contributed to the calculation of dietary pattern scores. To assess our sampling adequacy, we used the Kaiser-Mayer-Olkin statistic test which its result was 0.74 indicating a good appropriateness of factor analysis.

The PCA considers the correlations between food-groups intakes to identify underlying patterns in the data. PCA-derived dietary patterns were named using both quantitative and qualitative approaches [39]; in quantitative approach, the variable with the highest factor loading (e.g., fruits, vegetables, cereals, meat) or quantitative descriptions of dietary composition (e.g., high-fat or high-energy density) are considered, while in qualitative approach specific combinations of foods and/or descriptions of nutritional composition are considered [39]. Patterns that contained a variety of different foods or food groups that combined together in “more” and “less” healthy combinations are often given qualitative labels to denote healthfulness [39]. Accordingly, three following major dietary patterns were derived: 1) western-style dietary pattern: (high-fat/high-sodium/high-glycemic-index(GI) with a higher load of red meats, hydrogenated fats, sodium, and total fat intakes), 2) healthy pattern (with a higher load of whole grains, vegetables, and dairy products), and 3) processed-foods pattern (with a higher load of processed-meats, fast-foods, salty snakes, and sweets and candies). These dietary patterns explained 43.2% of the total variance in food intake (24.9, 9.6, and 8.7% of the patterns, respectively).

Factor scores of the participants were calculated using sum of multiplying the intake of the standardized food items by their respective factor loadings on each pattern.

### Definition of terms

Participants were categorized into different groups of glycemic status as follows [40, 41]: normal glycemia [i.e., normal fasting glucose (NFG) and normal glucose tolerance (NGT)], as FSG < 100 and 2-h serum glucose (2 h-SG) < 140; Pre-DM, as having at least one of the Impaired Fasting Glucose (IFG) (100 ≤ FSG < 126 mg/dL) or Impaired Glucose Tolerance (IGT) (140 ≤ 2 h-SG < 200 mg/dL); T2DM as FSG ≥ 126 mg/dL or 2 h-SG ≥ 200 mg/dL, or using glucose-lowering medications. A positive family history of T2DM was defined as having at least one parent or sibling with T2DM.

The T2DM-risk score was calculated as follows: SBP (mm Hg) < 120 (0 point), 120 < SBP < 140 (3 point), SBP ≥ 140 (7 point); family history of T2DM (5 point); waist-to-height ratio (WHR): < 0.54 (0 point), 0.54–0.59 (6 point), ≥ 0.59 (11 point); TG-to-HDL-C ratio: < 3.5 (0 point), ≥ 3.5 (3 point); FSG: < 90 mg/dL (0 point), ~ 90–99 mg/dL (12 point), ~ 100–125 mg/dL (33 point) [42].

### Statistical methods

Statistical analyses were conducted using the SPSS for Windows version 20 (SPSS Inc., Chicago, IL, USA). Baseline characteristics of the participants were compared across the groups using analysis of variance (ANOVA). The odds ratios (95% confidence intervals (CIs)) of Pre-DM regression to normal glycemia or progression to T2DM in relation to dietary pattern scores were estimated using multinomial logistic regression analysis. Potential covariates were selected based on both statistical and scientific evidence. A univariate analysis was performed for potential confounding variables, and those with *P*_E_ < 0.2 were selected for the final multivariable model; *P*_E_ (*P*-value for entry) determines which variables should be included in the multivariable model [43]. Finally, three logistic models, including crude model, adjusted-model 1 (adjusted for sex, age and T2DM-risk score), and adjusted-model 2 (additionally adjusted for body weight changes, smoking and physical activity) were conducted.

## Results

The mean age of the study participants was 49.4 ± 12.8 y, and 51.5% were men. During a median of 8.9 years of follow-up (inter-quartile range: 7.0-9.6 years), 133 cases (39.8%) of T2DM were diagnosed, 68 participants (20.4%) remained in Pre-DM state, and 133 participants (39.8%) backed into normal glycemia. The baseline characteristics of the study participants are summarized in Table 1. Participants who reversed to the normal glycemic state were significantly younger than those who remained at Pre-DM and or developed T2DM. The participants who developed T2DM had significantly higher FSG and 2 h-SG at baseline compared to other groups. Compared to T2DM patients, participants who backed into normal glycemia also had lower BMI and TG-to-HDL-C ratio at baseline. Other variables, i.e., blood pressures, smoking status, and physical activity levels, were comparable.

**Table 1:** Baseline characteristics of the study participants according to outcomes (*n* = 334)

	Normal glycaemia (*n* = 133)	Pre-DM (*n* = 68)	T2DM (*n* = 133)
Age *(y)*	45.2 ± 14.0 ^a,b^	51.5 ± 11.7	51.3 ± 11.2
Men *(%)*	48.9	55.9	51.9
FHD *(%)*	23.5	17.5	26.8
Current smoking *(%)*	14.3	14.9	13.7
Physical activity *(MET-h/week)*	26.0 ± 29.1	21.4 ± 23.6	23.6 ± 24.2
BMI *(kg/m*^*2*^*)*	28.9 ± 4.6	28.9 ± 4.5	29.5 ± 4.8
WC *(cm)*	96.3 ± 11.3	98.1 ± 10.1	100 ± 10.3
SBP *(mm Hg)*	119 ± 16.2	121 ± 16.8	124 ± 18.1
DBP *(mm Hg)*	77.0 ± 9.5	78.5 ± 11.8	79.6 ± 11.8
FSG *(mg/dL)*	98.2 ± 9.7^b^	101 ± 8.4^b^	105 ± 8.9
2 h-SG *(mg/dL)*	125 ± 30.0^b^	131 ± 35.6^b^	145 ± 31.3
TG to HDL-C ratio	4.3 ± 3.2^b^	5.2 ± 3.6	5.4 ± 3.8

Data are mean ± SD (unless stated otherwise)

^†^Median (inter-quartile range)

^a^significant difference with Pre-DM

^b^significant difference with T2DM

*T2DM* type 2 diabetes, *FHD* Family history of T2DM, *BMI* body mass index, *WC* waist circumference, *SBP* systolic blood pressure, *DBP* diastolic blood pressure, *FSG* fasting serum glucose, *2 h-SG* 2-h serum glucose, *TG* serum triglyceride, *HDL-C* high-density lipoprotein cholesterol; Normal glycemia, normal fasting glucose-normal glucose tolerance

The associations between dietary pattern scores and nutrient intakes are shown in Table 2. The Western-style dietary pattern was positively correlated with dietary fats and sodium, and negatively correlated with dietary fiber, protein and potassium intakes. Table 3 represents the odds ratio (95% CI) of Pre-DM regression to normal glycemia and progression to T2DM in relation to dietary pattern scores. Despite other patterns being not related to the chance of progression or regression of Pre-DM, the Western-style dietary pattern increased the risk of progressing to T2DM by 38% (OR = 1.36; 95% CI: 1.00 to 1.89, *P* = 0.050) independent of the well-known risk factors of T2DM.

**Table 2:** Correlation of dietary patterns scores with nutrients intakes

	DASH-style	MDS	western-style pattern	Healthy pattern	Processed-foods pattern
Total fat	-	-0.13	0.31	-	0.23
Saturated Fat	-	-0.29	0.21	0.25	-
MUFA	-	-	0.28	-	0.22
PUFA	-	-	0.32	-	0.19
Protein	-	-	-0.29	0.22	-
Sodium	-0.11	-0.11	0.25	-0.16	-
Potassium	0.18	0.11	-0.32	0.16	-
Total fiber	-	0.51	-0.15	-	-
Vitamin C	0.11	-	-0.22	-	-
Vitamin E	0.24	0.11	0.12	-	-
Calcium	0.17	-	-0.24	0.36	-

Data are coefficient r (only statistically significant values are shown in the table)

Partial correlation with adjustment for total energy intakes was used

*DASH-style* Dietary Approaches to Stop Hypertension, *MUFA* monounsaturated fatty acid, *PUFA* polyunsaturated fatty acid, *MDS* mediterranean diet score

**Table 3:** The odds ratio (95% CI) of Pre-DM regression to normal glycemia and progression to T2DM in relation to dietary pattern scores

Dietary patterns	Normal glycemia	T2DM
DASH-style		
Crude	0.97 (0.91–1.03)	1.02 (0.96–1.09)
Model 1	0.98 (0.91–1.05)	1.02 (0.96–1.09)
Model 2	0.98 (0.92–1.06)	1.03 (0.96–1.11)
MDS		
Crude	1.04 (0.86–1.25)	1.11 (0.92–1.34)
Model 1	1.05 (0.87–1.29)	1.11 (0.92–1.33)
Model 2	1.02 (0.86–1.29)	1.06 (0.86–1.29)
Western-style pattern		
Crude	1.08 (0.80–1.44)	1.32 (0.99–1.75)
Model 1	1.01 (0.98–1.03)	1.34 (1.00–1.78)^*^
Model 2	1.03 (0.75–1.41)	1.38 (1.00–1.89)^**^
Healthy pattern		
Crude	0.92 (0.69–1.21)	0.89 (0.68–1.18)
Model 1	0.90 (0.68–1.19)	0.89 (0.68–1.18)
Model 2	0.84 (0.63–1.13)	0.80 (0.59–1.08)
Processed-foods pattern		
Crude	1.13 (0.84–1.53)	1.16 (0.86–1.57)
Model 1	1.02 (0.74–1.40)	1.16 (0.85–1.58)
Model 2	1.04 (0.75–1.43)	1.17 (0.86–1.61)

Multinomial logistic regression was used

Model 1, adjusted for age, sex, T2DM-risk score

Model 2, additionally adjusted for weight-changes, smoking, and physical activity

^*^P = 0.050, ^**^P = 0.053

*Pre-DM* pre diabetes, *T2DM* type 2 diabetes mellitus, *Normal glycemia* normal fasting glucose-normal glucose tolerance, *MDS* Mediterranean diet score

## Discussion

In this 9-year follow-up of subjects with Pre-DM, the Western-style dietary pattern characterized by high amounts of red meats, hydrogenated fats, sodium intake, and high-GI food, increased the risk of Pre-DM progressing to T2DM by 38%. However, it did not show any significant relationship with returning to NGR. Other dietary pattern scores, i.e., DASH-style pattern, MDS, healthy-style pattern, and processed-foods pattern showed no significant association with either regression or progression of Pre-DM.

Dietary factors affecting the risk of developing T2DM are well documented [44–46]. However, the association of diet with Pre-DM regression and progression has been less investigated, and their results are inconclusive. Primary evidence suggested that low-calorie diets decreased the incidence of T2DM in people with IGT over six years [20], and a 40-g increase in dietary fats increased the risk of progression from IGT to T2DM to sixfold [47]. Other observational studies have concluded that high-GL diet accelerates the progression of Pre-DM by 85% [24] and 64% [48]. All of the mentioned studies have assessed the components of our western-style pattern (fats and high-GL diets). Our results support the previous findings: there is a positive association between the western-style pattern and risk of Pre-DM progression. Furthermore, in the present study, western-style pattern negatively correlates with protein and calcium, which are the main content of dairy products. In accordance with our result, a recent cohort has shown that decreasing low-fat dairy product consumption increased the risk of progression from Pre-DM to T2DM after 9 years of follow-up [23]. Also, contrary to our hypothesis, the western-style pattern did not affect the chance of returning to NGR.

Some possible mechanisms might underlie between western-style pattern characterized by high fat, high sodium, and high-GI foods in our study and the risk of T2DM. First, sodium intake influences the renin-angiotensin system activity [49], insulin resistance [50], catecholamine levels and lipids [51]. Each of these factors could be a potential mediator in the development of T2DM. Moreover, excessive sodium intake activates the sympathetic nervous system, which causes an increase in the peripheral vascular resistance, then promoting hypertension [52]. Second, dysregulation of fat metabolism happens in the primary steps of insulin resistance development. Free fatty acids (FFA) are independent predictors of progression to T2DM. There is a general agreement that elevated FFA flux from an expanded adipose tissue to non-adipose tissues has a deleterious effect on insulin regulation of carbohydrate metabolism. It is an important cause of the hypertriglyceridemia of T2DM, aggravates cytosolic triglyceride accumulation in non-adipose tissues, and may have other direct adverse effects, such as effects on endothelium, myocardium, and cell proliferation [53]. Last but not least, higher GI diets are positively associated with HbA1c concentrations, a result of higher plasma glucose levels, and in turn associated with lower plasma adiponectin concentrations. Adiponectin is associated with a lower risk of T2DM by several proposed mechanisms, including increasing insulin sensitivity and anti-inflammatory effects [54].

In previous studies, high adherence to the Med Diet has been associated with a 44% to 85% reduction in the risk of progression from Pre-DM to T2DM [21, 22]. The null association observed in our study may be due to the different approaches to calculate MDS [55], and the diverse characteristics of study populations, as well as the different study designs and follow-up time.

The relationship between diet and returning to NGR is less investigated by observational studies. Clinical trials have consistently shown the possibility of returning to NGR through combined drug therapy and lifestyle intervention (both diet and physical activity) [7] although most of them have not focused on the diet alone [26]. In contrast to a recent cohort which has found a significant relationship between the lower dietary GL, but not GI, and increased incidence of NGR [24], we did not find any significant relationship with neither of the patterns, even after controlling for confounders.

This study had some strength. First, this study is a longitudinal cohort of Pre-DM subjects among an Asian population with a higher prevalence of T2DM. Second, we used a valid and reliable collection of the dietary data using a semi-quantitative 168-FFQ that reduced the possibility of reporting biases. Third, the well-known risk factors of T2DM were detected and controlled in our analyses, however, due to existing of other possible unknown risk factors, complete controlling for confounders was not possible in our models. Using PCA to derived dietary pattern scores, as a holistic analytical approach that reflects the complexity of the human diet and measures cumulative and interactive effects dietary exposures in a sample population, was also strength. Using multinomial logistic regression, enabled us to include the exposure and three-categorized outcome variables (i.e., normal glycemia, Pre-DM, and T2DM), improves the study power and provided us the chance of comparing Odds of the outcomes simultaneously. Finally, previous studies have defined Pre-DM by just one factor such as IGT, however our study included both IGT and IFG criteria.

## Conclusions

In conclusion, we found a positive association between western-style dietary pattern (with higher load of red meats, hydrogenated fats, sodium and high-GI foods intakes) with progressive Pre-DM to T2DM over a 9-years of follow-up.

## Supplementary Information

**Additional file 1.**

## Data Availability

The datasets generated and/or analysed during the current study are not publicly available due to the ethical concerns but are available from the corresponding author on reasonable request.

## Abbreviations

Pre-DM: Pre-diabetes
T2DM: Type2 Diabetes Mellitus
CI: Confidence Interval
OR: Odds Ratio
Med Diet: Mediterranean diet
GL: Glycemic Load
GI: Glycemic Index
NGR: Normal Glucose Regulation
MDS: Mediterranean Diet Score
TLGS: Tehran Lipid and Glucose Study
WC: Waist Circumference
BMI: Body Mass Index
SBP: Systolic Blood Pressure
DBP: Diastolic Blood Pressure
FSG: Fasting Serum Glucose
2 h-SG: 2-Hour Serum Glucose
IFG: Impaired Fasting Glucose
IGT: Impaired Glucose Tolerance
NFG: Normal Fasting Glucose
NGT: Normal Glucose Tolerance
TG: Triglyceride
HDL-c: High-Density Lipoprotein cholesterol
OGTT: Oral Glucose Tolerance Test
MAQ: Modifiable Activity Questionnaire
FFQ: Food Frequency Questionnaire
DASH: Dietary Approaches to Stop Hypertension
MUFAs to SFAs ratio: Dietary Monounsaturated-to-Saturated fats ratio
PCA: Principal Component Analysis
