# Supplementary material for: Dietary pattern scores in relation to pre-diabetes regression to normal glycemia or progression to type 2 diabetes: a 9-year follow-up
Source: BMC Endocr Disord. 2023 Jan 20;23:20. doi: 10.1186/s12902-023-01275-9 (PMC9854100; doi:10.1186/s12902-023-01275-9)
Supplement: Supplementary file 1 — Additional file 1. [file 12902_2023_1275_MOESM1_ESM.docx]

Supplementary file 1

Research Institute of Endocrine Sciences

Shaheed Beheshti University of Medical Sciences

Food Frequency Questionnaire (FFQ) "Tehran Lipid and Glucose Study"

Name: Acceptance Code: Age:

| NO. | DAIRY FOODS | HOW MUCH |  | HOW | OFTEN |  | CONSIDERATIONS |
| --- | --- | --- | --- | --- | --- | --- | --- |
|  |  |  | per day | per week | per month | per year |  |
| 1 | Lavashbread(refined grains) | 1 loaf |  |  |  |  |  |
| 2 | Barbari bread(refined grains) | 1 loaf |  |  |  |  |  |
| 3 | Sangak bread(whole grains) | 1 loaf |  |  |  |  |  |
| 4 | Taftoon bread(refined grains) | 1 loaf |  |  |  |  |  |
| 5 | Baguette bread | 1 loaf |  |  |  |  |  |
| 6 | toast bread(whole grains) | 1 loaf |  |  |  |  |  |
| 7 | Cooked rice | 1 dinner plate |  |  |  |  | normal: full: |
| 8 | Cooked pasta | 1 spatula |  |  |  |  | Flat head: full: |
| 9 | Potato | 1 medium |  |  |  |  |  |
| 10 | French fries | 1 slice |  |  |  |  |  |
| 11 | Baked vermicelli (soup noodle) | 1 cup |  |  |  |  |  |
| 12 | Ash noodle | 1 cup |  |  |  |  |  |
| 13 | wheat flour | 1 cup |  |  |  |  |  |
| 14 | Cookies(stating the type) | 1 number |  |  |  |  | type: |
| 15 | Crackers(crispy biscuits) | 1 number |  |  |  |  |  |
| 16 | Yazdi cake | 1 number |  |  |  |  |  |
| 17 | Homemade cake(birthday cake and ...) | 1 medium slice |  |  |  |  |  |
| 18 | Other cakes | 1 number |  |  |  |  |  |
| 19 | Corn | 1 medium |  |  |  |  |  |
| 20 | Barley | 1 tsp |  |  |  |  |  |
| 21 | Cooked barley or bulgur | 1 cup |  |  |  |  |  |
| 22 | Lentil | 1 cup |  |  |  |  |  |
| 23 | Beans | 1 cup |  |  |  |  |  |
| 24 | Pea | 1 cup |  |  |  |  |  |
| 25 | Baked bean | 1 cup |  |  |  |  |  |
| 26 | Soy bean | 1 cup |  |  |  |  |  |
| 27 | Mung | 1 cup |  |  |  |  |  |
| 28 | Cotyledon | 1 cup |  |  |  |  |  |
| 29 | Beef or calf | 1 slice of stewed |  |  |  |  |  |
| 30 | lamb meat | 1 slice of stewed |  |  |  |  |  |
| 31 | Ground beef | 1 tablespoon |  |  |  |  |  |
| 32 | Hen and Chickens with skin | 1 medium piece |  |  |  |  | Drumstick: chest: wings: with peel: peeled: |
| 33 | Hen and Chickens without skin | 1 medium piece |  |  |  |  |  |
| 34 | Fish (except tuna) stating the type | 1 medium piece (one palm full) |  |  |  |  | The amount of: type: |
| 35 | Tuna (canned) | 1/2 cans |  |  |  |  | Is it discarded oil? Yes * No * |
| 36 | Heart, liver and kidney | 1 skewer |  |  |  |  |  |
| 37 | Hamburger | 1 number |  |  |  |  |  |
| 38 | kielbasa | 1 Cut |  |  |  |  |  |
| 39 | Sausage | 1 number (Germany * cocktails *) |  |  |  |  |  |
| 40 | offal | 1 medium piece |  |  |  |  |  |
| 41 | Egg | 1 Number |  |  |  |  |  |
| 42 | Tripe and Rennet | 1 piece |  |  |  |  |  |
| 43 | Tongue | 1 whole number |  |  |  |  |  |
| 44 | Brain | 1 whole number |  |  |  |  |  |
| 45 | Head | 1 palm |  |  |  |  |  |
| 46 | Leg | 1 Number |  |  |  |  | If certain amount and components is used, noted. |
| 47 | Pizza | 1 number |  |  |  |  |  |
| 48 | Low-fat milk (less than 2%) | 1 cup |  |  |  |  |  |
| 49 | Whole milk (greater or equal to 2%) | 1 cup |  |  |  |  |  |
| 50 | Cacao milk | 1 cup |  |  |  |  |  |
| 51 | Chocolate milk | 1 cup |  |  |  |  |  |
| 52 | Ooze Yogurt | 1 tablespoon |  |  |  |  |  |
| 53 | Regular yogurt | 1 Bowl/cup |  |  |  |  |  |
| 54 | Full fat yogurt | 1 Bowl/cup |  |  |  |  |  |
| 55 | Yogurt cream | 1 tsp |  |  |  |  |  |
| 56 | Cheese | 1 pat |  |  |  |  |  |
| 57 | Cream cheese | 1 pat |  |  |  |  |  |
| 58 | Dough | 1 cup |  |  |  |  |  |
| 59 | Cream | 1 tablespoon |  |  |  |  |  |
| 60 | Traditional ice cream | Half cup |  |  |  |  | how months of year? |
| 61 | Non-traditional ice cream | 1 number |  |  |  |  | how months of year? |
| 62 | Butter | 1 pat |  |  |  |  |  |
| 63 | Margarine | 1 pat |  |  |  |  |  |
| 64 | Dried whey | 1 tablespoon |  |  |  |  |  |
| 65 | Shredded lettuce | 1 cup |  |  |  |  |  |
| 66 | Tomato | 1 medium |  |  |  |  |  |
| 67 | Cucumber | 1 medium |  |  |  |  |  |
| 68 | Fresh Herbs | 1 small plate |  |  |  |  |  |
| 69 | Cooked vegetables (soup, rice, etc.) | 1 cup |  |  |  |  |  |
| 70 | Pumpkin | 1 medium |  |  |  |  |  |
| 71 | Stewed pumpkin | 1 medium |  |  |  |  |  |
| 72 | Baked Eggplant | 1 medium |  |  |  |  |  |
| 73 | Boiled Celery | 1 cup |  |  |  |  |  |
| 74 | Green peas cooked | 1 cup |  |  |  |  |  |
| 75 | Green beans cooked | 1 cup |  |  |  |  |  |
| 76 | Raw carrots | 1 medium |  |  |  |  |  |
| 77 | Cooked carrots | 1 medium |  |  |  |  |  |
| 78 | Garlic | 1 clove |  |  |  |  |  |
| 79 | Raw onion | 1 small |  |  |  |  |  |
| 80 | Fried onions | 1 tablespoon |  |  |  |  |  |
| 81 | Cabbage varieties | 1 Bowl/cup |  |  |  |  |  |
| 82 | Bell peppers | 1 medium |  |  |  |  |  |
| 83 | Row spinach | 20 medium leaf |  |  |  |  |  |
| 84 | Cooked spinach | 1 cup |  |  |  |  |  |
| 85 | Turnip | 1 medium |  |  |  |  |  |
| 86 | Small green pepper | 1 medium |  |  |  |  |  |
| 87 | ketchup | 1 tablespoon |  |  |  |  |  |
| 88 | Pickles | 1 cup |  |  |  |  | how months of year? |
| 89 | SHOOR | 1 cup |  |  |  |  | how months of year? |
| 90 | Pickled cucumber | 1 medium |  |  |  |  |  |
| 91 | Cantaloupe | 1/4 number |  |  |  |  |  |
| 92 | Melon | 1 medium slice |  |  |  |  |  |
| 93 | Watermelon | 1 medium slice |  |  |  |  |  |
| 94 | Pear | 1 medium |  |  |  |  |  |
| 95 | Apricot | 1 medium |  |  |  |  |  |
| 96 | Cherries | 1 small plate |  |  |  |  |  |
| 97 | Apple | 1 medium |  |  |  |  |  |
| 98 | Peach | 1 medium |  |  |  |  |  |
| 99 | Nectarines | 1 medium |  |  |  |  |  |
| 100 | Prunus | 1 medium |  |  |  |  |  |
| 101 | Fresh figs | 1 medium |  |  |  |  |  |
| 102 | Dried figs | 1 medium |  |  |  |  |  |
| 103 | Grape | 1 medium bunch |  |  |  |  |  |
| 104 | Kiwi | 1 medium |  |  |  |  |  |
| 105 | Grapefruit | 1 medium |  |  |  |  |  |
| 106 | Orange | 1 medium |  |  |  |  |  |
| 107 | Persimmon | 1 medium |  |  |  |  |  |
| 108 | Tangerine | 1 medium |  |  |  |  |  |
| 109 | Pomegranate | 1 medium |  |  |  |  |  |
| 110 | Date | 1 medium |  |  |  |  |  |
| 111 | Plums (yellow and red) | 1 medium |  |  |  |  |  |
| 112 | Cherry | 10 medium |  |  |  |  |  |
| 113 | Strawberry | 1 number |  |  |  |  |  |
| 114 | Banana | 1 medium |  |  |  |  |  |
| 115 | Sweet lemon | 1 medium |  |  |  |  |  |
| 116 | Lemon | 1 medium |  |  |  |  |  |
| 117 | Grapefruit juice | 1 cup |  |  |  |  |  |
| 118 | Orange juice | 1 cup |  |  |  |  |  |
| 119 | Apple juice | 1 cup |  |  |  |  |  |
| 120 | Cantaloupe juice | 1 cup |  |  |  |  |  |
| 121 | Cornelian cherry | 1 cup |  |  |  |  |  |
| 122 | Fresh Pineapple | 1 cup |  |  |  |  |  |
| 123 | Canned Pineapple | 1 cup |  |  |  |  |  |
| 124 | Raisins | 1 tablespoon |  |  |  |  |  |
| 125 | Cantaloupe |  |  |  |  |  |  |
| 126 | Fresh berries | 1 small plate |  |  |  |  |  |
| 127 | Dried berries | 1 number |  |  |  |  |  |
| 128 | dried Peach | 10 number |  |  |  |  |  |
| 129 | dried apricot | 10 number |  |  |  |  |  |
| 130 | Green Olive | 1 number |  |  |  |  |  |
| 131 | Canned fruits | 1 can |  |  |  |  |  |
| 132 | Solid vegetables oils | 1 tablespoon |  |  |  |  |  |
| 133 | Oil | 1 tablespoon |  |  |  |  |  |
| 134 | Olive oil | 1 tablespoon |  |  |  |  |  |
| 135 | suset | 1 medium slice |  |  |  |  |  |
| 136 | Ghee | 1 tablespoon |  |  |  |  |  |
| 137 | Mayonnaise | 1 tablespoon |  |  |  |  |  |
| 138 | Peanut | 1 number |  |  |  |  |  |
| 139 | Almond | 1 number |  |  |  |  |  |
| 140 | Walnut | 1whole number |  |  |  |  |  |
| 141 | Pistachios | 1 number |  |  |  |  |  |
| 142 | Hazelnut | 1 number |  |  |  |  |  |
| 143 | Seeds (watermelon, pumpkin, sunflower) | 1 Bowl/cup |  |  |  |  |  |
| 144 | Sugar cube, comfit | 1 number |  |  |  |  |  |
| 145 | Sugar | 1 teaspoonful |  |  |  |  |  |
| 146 | Honey | 1 teaspoonful |  |  |  |  |  |
| 147 | Jams (by type) | 1 tablespoon |  |  |  |  |  |
| 148 | Industrial Or cola beverages. | 1 cup |  |  |  |  |  |
| 149 | Dried sweets | 1 medium |  |  |  |  |  |
| 150 | Cream Sweets | 1 medium |  |  |  |  |  |
| 151 | GAZ | 1 medium |  |  |  |  |  |
| 152 | candy | 1 number |  |  |  |  |  |
| 153 | SOHAN | 1 Piece |  |  |  |  |  |
| 154 | Puff | 1 pack |  |  |  |  |  |
| 155 | Chocolate | 1 number |  |  |  |  |  |
| 156 | caramel cream | 1 tsp |  |  |  |  |  |
| 157 | Tea | 1 cup |  |  |  |  |  |
| 158 | Salt | 1 tsp |  |  |  |  | Type: |
| 159 | Broth(only water) | 1 cup |  |  |  |  |  |
| 160 | Chips | 1 pack |  |  |  |  |  |
| 161 | Coffee | 1 cup |  |  |  |  |  |
| 162 | Lemon juice | 1 teaspoonful |  |  |  |  |  |
| 163 | Candy | 1 medium |  |  |  |  |  |
| 164 | Baked mushrooms | Half cup |  |  |  |  |  |
| 165 | Homemade HALVA | 1 tablespoon |  |  |  |  |  |
| 166 | Sesame pudding | 1 tablespoon |  |  |  |  |  |
| 167 | Noghl | 10 number |  |  |  |  |  |
| 168 | Donuts | 1 number |  |  |  |  |  |

The date of delivery of food questionnaires to residents:

Name of dietitian responsible for completing FFQ:

Delivery date to Nutrition expert:
